# Mental health of healthcare workers during the first year of the COVID-19 pandemic in the Netherlands: a longitudinal study

**DOI:** 10.3389/fpubh.2023.1224112

**Published:** 2023-11-23

**Authors:** Maaike van der Noordt, Karin I. Proper, Bette Loef, Cécile R. L. Boot, Floor M. Kroese, Marijn de Bruin, Sandra H. van Oostrom

**Affiliations:** ^1^Department of Public Health Foresight, Center for Health and Society, National Institute for Public Health and the Environment (RIVM), Bilthoven, Netherlands; ^2^Department Behaviour & Health, Center for Prevention, Lifestyle and Health, National Institute for Public Health and the Environment (RIVM), Bilthoven, Netherlands; ^3^Department of Public and Occupational Health, Amsterdam Public Health Research Institute, Amsterdam UMC, VU University Amsterdam, Amsterdam, Netherlands; ^4^Societal Participation and Health, Amsterdam Public Health Research Institute, Amsterdam, Netherlands; ^5^Work, Health and Performance, Behavioural Science Institute, Radboud University, Nijmegen, Netherlands; ^6^Department of Social, Health, and Organizational Psychology, Utrecht University, Utrecht, Netherlands; ^7^IQ Healthcare, Institute of Health Sciences, Radboud UMC, Nijmegen, Netherlands

**Keywords:** healthcare workers, mental health, COVID-19, longitudinal study, occupational health

## Abstract

**Purpose:**

In March 2020, the WHO declared COVID-19 a pandemic. Previous virus outbreaks, such as the SARS outbreak in 2003, appeared to have a great impact on the mental health of healthcare workers. The aim of this study is to examine to what extent mental health of healthcare workers differed from non-healthcare workers during the first year of the COVID-19 pandemic.

**Methods:**

We used data from a large-scale longitudinal online survey conducted by the Corona Behavioral Unit in the Netherlands. Eleven measurement rounds were analyzed, from April 2020 to March 2021 (*N* = 16,615; number of observations = 64,206). Mental health, as measured by the 5-item Mental Health Inventory, was compared between healthcare workers and non-healthcare workers over time, by performing linear GEE-analyses.

**Results:**

Mental health scores were higher among healthcare workers compared to non-healthcare workers during the first year of the pandemic (1.29 on a 0–100 scale, 95%-CI = 0.75–1.84). During peak periods of the pandemic, with over 100 hospital admissions or over 25 ICU admissions per day and subsequently more restrictive measures, mental health scores were observed to be lower in both healthcare workers and non-healthcare workers.

**Conclusion:**

During the first year of the COVID-19 pandemic, we observed no relevant difference in mental health between healthcare workers and non-healthcare workers in the Netherlands. To be better prepared for another pandemic, future research should investigate which factors hinder and which factors support healthcare workers to maintain a good mental health.

## Introduction

In March 2020, the World Health Organization declared COVID-19 a pandemic (1). Previous virus outbreaks such as the Severe Acute Respiratory Syndrome (SARS) outbreak in 2003, demonstrated a great impact on the mental health of healthcare workers (2–5). Several factors were identified to explain higher levels of distress among healthcare workers during the SARS-outbreak, including fear of infection, social isolation, and job stress (5). With the severity of the coronavirus SARS-CoV-2 and the uncertainty caused by it, it is plausible to expect an impact of the COVID-19 pandemic on the mental health of healthcare workers (6, 7). Insight in the mental health of healthcare workers during the COVID-19 pandemic is needed to provide recommendations for healthcare workers, employers and policy makers to maintain good health and employability of healthcare workers during a pandemic.

Two systematic reviews showed that healthcare workers reported high levels of depression, anxiety, insomnia and distress early in the COVID-19 pandemic (8, 9). A meta-analysis showed a pooled prevalence of 22.8% for depression, 23.2% for anxiety and 38.9% for insomnia during the first months of the pandemic among healthcare workers (9). In contrast, the prevalence of depression before the pandemic was 7.0% among the working-age population in the European Union in 2019 (10). Risk factors identified for mental health problems in this pandemic situation were inadequate personal protective equipment, close contact with COVID-19 patients, heavy workload, being female and underlying illness (8). The studies included in the reviews focused mainly on “frontline” hospital workers, i.e., those working directly with COVID-19 patients. It can be expected that other healthcare workers also experience more stress than non-healthcare workers, as most of them come in close contact with patients, leading to increased risk of getting infected (11). Some may fear to get ill themselves, others to become the source of infection to their loved ones, i.e., family members who are older, immunocompromised, or chronically ill. This fear may lead to excessive stress and mental health problems (12, 13).

When comparing mental health between healthcare workers and non-healthcare workers, studies showed that healthcare workers actually appeared to have similar, or even lower prevalence of stress, anxiety, depression and post-traumatic stress disorder compared to non-healthcare workers during the first wave of the pandemic (14–17). The main explanations for these findings were that healthcare workers feel better informed about the virus and measures to avoid getting infected, and better understand why these measures are needed (14). Moreover, it is noted that (frontline) healthcare workers have access to formal psychological support, in contrast to non-healthcare workers (15). Finally, they suggest that healthcare workers were less exposed to lockdown measures such as social distancing, and economic instability (17).

So far, most research examined the mental health of healthcare workers cross-sectionally during the first wave of the pandemic (8, 9). However, there are indications that different points in the outbreak curve have affected mental health differently (8). As infections rise, the pressure on the healthcare workers rises as well, and measures to prevent the virus from spreading become more restrictive (18–20). It is therefore of interest to assess the mental health status of healthcare workers over time. To our knowledge, only one study examined mental health of (Finnish) hospital workers longitudinally from June to November 2020. It shows that mental health of the hospital workers fluctuated, and was associated with the number of infections and subsequent restrictive measures (21). In Finland, the pressure on healthcare workers in this study period was not as high as in other countries and risk of infection was relatively low, due to a limited number of COVID-19 cases (22). Our study focusses on the first full year of the pandemic in the Netherlands, a country that experienced peak levels of the pandemic (i.e., the highest risk level of >100 hospital admissions/day or > 25 ICU admissions/day) from March 18 to April 23 (2020) and from September 28 (2020) to May 26 (2021) (18).

The aim of this study is to examine to what extent mental health of healthcare workers differed from non-healthcare workers during the COVID-19 pandemic, using longitudinal data collected in the Netherlands during the first year of the pandemic (April 2020 until March 2021).

## Methods

### Study design

From the start of the COVID-19 pandemic in the Netherlands a large-scale longitudinal online survey (Corona Behavioral Unit (CBU) Cohort; first wave April 2020) was carried out by the Dutch National Institute for Public Health and the Environment in collaboration with the Association of Municipal Health Services and Regional Medical Emergency Preparedness and Planning offices in the Netherlands and 25 Municipal Health Services (23). Participants of pre-existing panels of the Municipal Health Services (*n* = 1,000 to 10,000 per panel) were invited to participate in the cohort study. Participants of the various panels were recruited in different ways, including random sample selection, through specific ongoing studies or via (social) media. The first questionnaire was sent out on April 17, 2020, followed by additional questionnaires every 3 weeks. After round 5, the frequency of the questionnaires was reduced to a six-week cycle. From round 3 onwards, the cohort became a “dynamic cohort,” as new participants could enter the survey in rounds 3, 5, 6, 8, and 10. Participants were additionally recruited via social media and various mailing networks (e.g., of higher education organizations), in order to recruit additional participants who were underrepresented in the cohort (e.g., young people). To limit the questionnaire length, participants were randomly assigned to one of three groups upon entering the cohort. Each group received different blocks of questions and there was a one-time crossover of blocks after enrollment. To specify with regard to mental health, one of the three groups received questions about mental health only during enrollment. Another group received questions about mental health in all the subsequent rounds. The third group never received any questions about mental health and was therefore not included in the analyses of the present study. The CBU cohort study does not meet the requirement as laid down in the Law for Research Involving Human Subjects (WMO) and was therefore exempted by the Centre for Clinical Expertise at RIVM from formal ethical review (Study number G&M-561).

### Study sample

**Figure 1:**
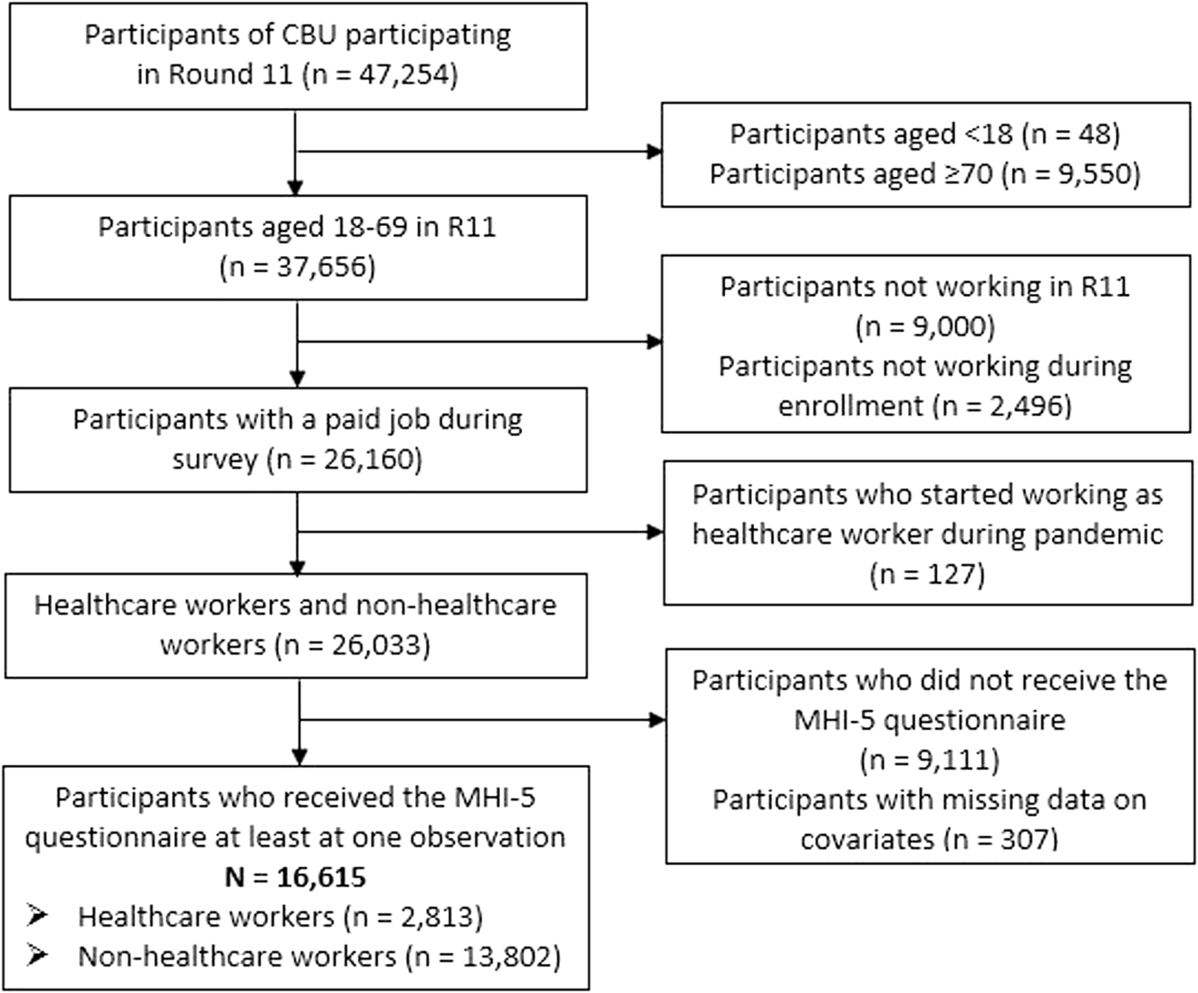
Flowchart of the selection of the study sample.

Data from round 1 (17–24 April 2020) to round 11 (24–28 March 2021) were analyzed. Data from round 11 were used for selection of participants for our study as questions about being a healthcare worker or non-healthcare worker were only asked in round 11. In total, 47,254 people participated in round 11 of the CBU Cohort. Of those, participants aged 18–69 years who had a job in round 11 and also during enrollment in the study were selected (*n* = 26,160). Participants were excluded if they started to work as a healthcare worker during the pandemic (*n* = 127), because it was unknown when they had exactly started. Subsequently, the subgroup of participants that did not receive questions on mental health (based on block randomization, see Study design) was excluded (*n* = 9,111). Participants with missing data on selected covariates (e.g., sex, age, education) were also excluded (*n* = 307). Finally, 16,615 participants remained in the sample for the analyses, who yielded a total of 64,206 observations. See the flowchart in Figure 1 for details. All participants participated at least two times in the survey: during enrollment and in round 11. Due to the use of different blocks of questions with a one-time cross-over, participants could contribute 1–10 observations of the 11 rounds. Of the 16,615 participants selected for this study, 8,754 contributed one observation, 5,770 participants contributed 2 to 9 observations and 2,091 participants contributed 10 observations to the analyses.

### Measures

#### Work

To distinguish healthcare workers from non-healthcare workers, the following questions were used. First: “In which occupational sector do you work?” Fourteen response categories were given, listing 13 occupational sectors and one answer category: “I do not work/I am retired.” Respondents who indicated to work in the occupational sector “Healthcare/welfare” were subsequently asked whether they were healthcare workers. Response categories were: 1. “Yes, I was a healthcare worker before the pandemic and I still am,” 2. “Yes, I am a healthcare worker since the first wave of the pandemic (March 2020–Juni 2020),” 3. “Yes, I am a healthcare worker since the second wave of the pandemic (July 2020–March 2021),” or 4. “No.” Only respondents who answered “Yes, I was a healthcare worker before the pandemic and I still am” (response category 1) were categorized as a “healthcare worker.” Respondents who answered to start working as a healthcare worker during the pandemic (response category 2 or 3) were excluded from the sample (view paragraph “study sample”). Respondents who answered “No” (response category 4), and those who indicated they were working in one of the other 12 sectors were categorized as “non-healthcare workers.”

Respondents who indicated they were healthcare workers were also asked to indicate their workplace: 1. Hospital, 2. Nursing home, care home or hospice, 3. General practice, 4. Home care, 5. Mental healthcare, or 6. Other healthcare setting.

#### Mental health

Mental health was measured with an adapted version of the Mental Health Inventory (MHI-5). The MHI-5 measures general mental health (24) and is part of the 36-item Short Form Health Survey, a questionnaire for measuring health-related quality of life (25). The MHI-5 contains the following items: “How much of the time during the last 4 weeks have you: (i) been a very nervous person?; (ii) felt so down in the dumps that nothing could cheer you up?; (iii) felt calm and peaceful?; (iv) felt downhearted and blue?; and (v) been a happy person?” For each question response categories were: 1. all of the time, 2. most of the time, 3. a good bit of the time, 4. some of the time, 5. a little of the time, or 6. none of the time. Because items (iii) and (v) ask about positive feelings, their scoring was reversed. Instead of referring to *the last four weeks*, the adapted version referred to *the last week*, because questionnaires were initially send out every 3 weeks. The mean MHI-5 score was computed by summing up the scores of each item and then multiplying the raw scores by 4, to transform it into a 0–100-point scale. A score of 100 represents optimal mental health (25). The mean scores are reported.

#### Covariates

Various categories of covariates were included: demographics (age, sex and educational level), health (health condition and past suspected/confirmed COVID-19 infection) and social environment (household composition and quality of social contacts). The demographic covariates were fixed variables. Health and social environment variables were time-varying variables derived from each individual round. See Supplementary Appendix for details on the construction of these variables.

### Statistical analysis

Differences between healthcare workers and non-healthcare workers in the study sample were tested using chi-square tests and one Mann–Whitney-test regarding the MHI-5 scores.

Four linear Generalized Estimating Equations (GEE) analyses were performed to test the associations between working in health care and general mental health during the pandemic. The crude model (model 1) was adjusted step-by-step for demographic variables (model 2), health variables (model 3), and social environment variables (model 4). Since the distributions of the MHI-5 scores and its residuals were negatively skewed, a cross-validation analysis was performed using a square root transformation of the MHI-5 scores to test the validity of the results (26).

Next, to examine whether the mental health trajectories over time differed between healthcare workers and non-healthcare workers, an adjusted linear GEE-analysis was performed in which measurement round was also included in the model as factor. Reported means were adjusted for all covariates. The mental health trajectories for healthcare workers and non-healthcare workers were plotted in a graph and compared to the risk levels (18).

Finally, to examine differences in mental health between healthcare workers working at different workplaces, a linear GEE-analysis was performed among health care workers with healthcare setting as main determinant and mental health as outcome. Statistical differences were tested using 95% confidence intervals. This analysis was also adjusted for all covariates.

Analyses were conducted using IBM SPSS Statistics, version 24.0.

## Results

### Study population

Table 1 shows the characteristics of healthcare workers and non-healthcare workers in Round 11. It shows that the composition of the groups differ in demographic characteristics (sex, age, and educational level) and household composition. For example, healthcare workers were more often female (90.4% vs. 66.5%) and less often highly educated (60.4% vs. 69.7%), compared to the non-healthcare workers. In addition, healthcare workers reported more often a suspected/confirmed infection by COVID-19 during the first year of the pandemic (22.9% vs. 17.7%), which could reflect infection risk but also differences in the availability of COVID-19 testing facilities.

**Table 1:** Characteristics of the study population stratified for healthcare workers and non-healthcare workers during the COVID-19 pandemic (round 11—March 2021) (*n* = 16,615).

	Healthcare workers (*n* = 2,813)		Non-healthcare workers (*n* = 13,802)	
	*%*	*n*	*%*	*n*
Sex (% female)*	90.4	2,544	66.5	9,173
Age*				
18-29	5.8	163	6.4	885
30–39	23.1	651	19.0	2,622
40–49	25.2	710	27.6	3,808
50–59	29.5	830	30.0	4,134
60–69	16.3	459	17.0	2,353
Educational level (%)*				
Low	5.0	141	5.5	756
Middle	34.8	978	24.8	3,429
High	60.2	1,694	69.7	9,617
Health condition (% yes)	18.7	525	17.4	2,396
Suspected or confirmed COVID-19 infection (% yes)*	23.1	649	17.8	2,452
Household composition (%)*				
Living alone	12.2	343	14.1	1938
Living with partner	27.8	781	31.8	4,360
Living with children ≤ 12 years	31.6	887	29.5	4,052
Living with children > 12	25.3	711	21.2	2,907
Living with others	3.0	83	3.4	460
Missing		8		85
Quality of social contacts (%)				
Not good	21.5	306	23.1	1,626
Neutral	28.4	404	27.5	1936
Good	50.2	715	49.4	3,478
Missing^a^		1,388		6,762
Healthcare setting (%)				
Hospital	17.9	504		
Nursing home	16.5	464		
General practice	5.8	163		
Homecare	11.2	316		
Mental healthcare	10.6	297		
Other healthcare setting	38.0	1,069		
Occupational sector				
Agricultural			0.8	110
Business/administrative			9.7	1,335
Commercial			6.5	891
Creative/linguistic			3.4	465
Services			11.3	1,559
IT			7.1	986
Managers			4.7	647
Public administration/security/legal			11.6	1,597
Educational			10.2	1,413
Technical			5.8	802
Transport/logistics			2.7	370
Healthcare (other than healthcare worker)			7.5	1,035
Other			18.8	2,592

	Mean	SD	Mean	SD
Mental health (mean MHI-5 score)	74.0	16.8	73.3	17.2
Missing (n)^a^		1,388		6,762

*Characteristic differs significantly between healthcare workers and non-healthcare workers (*p* < 0.05). ^a^Variable information in round 11 only available for a subsample, i.e., the respondents assigned to the module with questions about mental health.

### Mental health

The crude linear regression analysis shows that the mean MHI-5 score in the period from April 2020 to March 2021 was 0.62 (95%-CI = 0.03–1.21) points higher among healthcare workers compared to non-healthcare workers (Table 2; model 1). Higher scores on the 0–100 scale indicate better mental health. After adjusting for demographic factors, mean scores differed slightly more (B = 1.50; 95%-CI = 0.90–2.09) (model 2). After adjusting for all potential confounders, the average mental health of healthcare workers was 1.29 (95%-CI = 0.75–1.84) points higher compared to non-healthcare workers (model 4). The adjusted mean MHI-5 scores in this final model were 71.5 among healthcare workers and 70.3 among non-healthcare workers. Square root transformation of the MHI-5 scores confirmed these findings as no substantial difference between health-care workers and non-healthcare workers were found. After back-transformation the effect estimate of the fully adjusted model was 1.27 points.

**Table 2:** Effect estimates of the association between healthcare worker and mental health (difference in mean MHI-5 score).

		Model 1		Model 2		Model 3		Model 4
	B	95% CI	B	95% CI	B	95% CI	B	95% CI
Healthcare worker (yes vs. no)	0.62	0.03; 1.21	1.50	0.90; 1.09	1.57	0.97; 2.16	1.29	0.75; 1.84
Sex (male vs. female)			2.89	2.37; 3.40	2.90	2.39; 3.42	3.47	3.00; 3.94
Age								
18–29			−12.07	−13.23; −10.90	−11.93	−13.09; −10.78	−10.30	−11.42; −9.19
30–39			−5.52	−6.27; −4.76	−5.40	−6.15; −4.64	−5.57	−6.35; −4.79
40–49			−2.88	−3.55; −2.21	−2.76	−3.43; −2.09	−3.33	−4.04; −2.63
50–59			−1.89	−2.53; −1.25	−1.71	−2.34; −1.07	−1.74	−2.36; −1.13
60–69 (ref)								
Educational level								
Low			−1.29	−2.33; −0.24	−1.13	−2.17; −0.09	−0.95	−1.90; 0.00
Middle			−0.94	−1.46; −0.42	−0.84	−1.36; −0.32	−0.66	−1.14; −0.19
High (ref)								
Health condition (yes vs. no)					1.89	1.46; 2.32	1.70	1.29; 2.10
Suspected or confirmed COVID-19 infection (yes vs. no)					−1.81	−2.23; −1.40	−1.62	−2.01; −1.22
Household composition								
Living alone							−3.37	−4.05; −2.68
Living with children ≤ 12 years							1.18	0.54; 1.82
Living with children > 12							0.29	−0.30; 0.88
Living with others							−2.81	−4.20; −1.41
Living with partner (ref)								
Quality of social contacts								
Good							9.41	9.06; 9.75
Neutral							4.89	4.56; 5.21
Not good (ref)								

B, beta; CI, confidence interval.

### Trend in mental health over time

**Figure 2:**
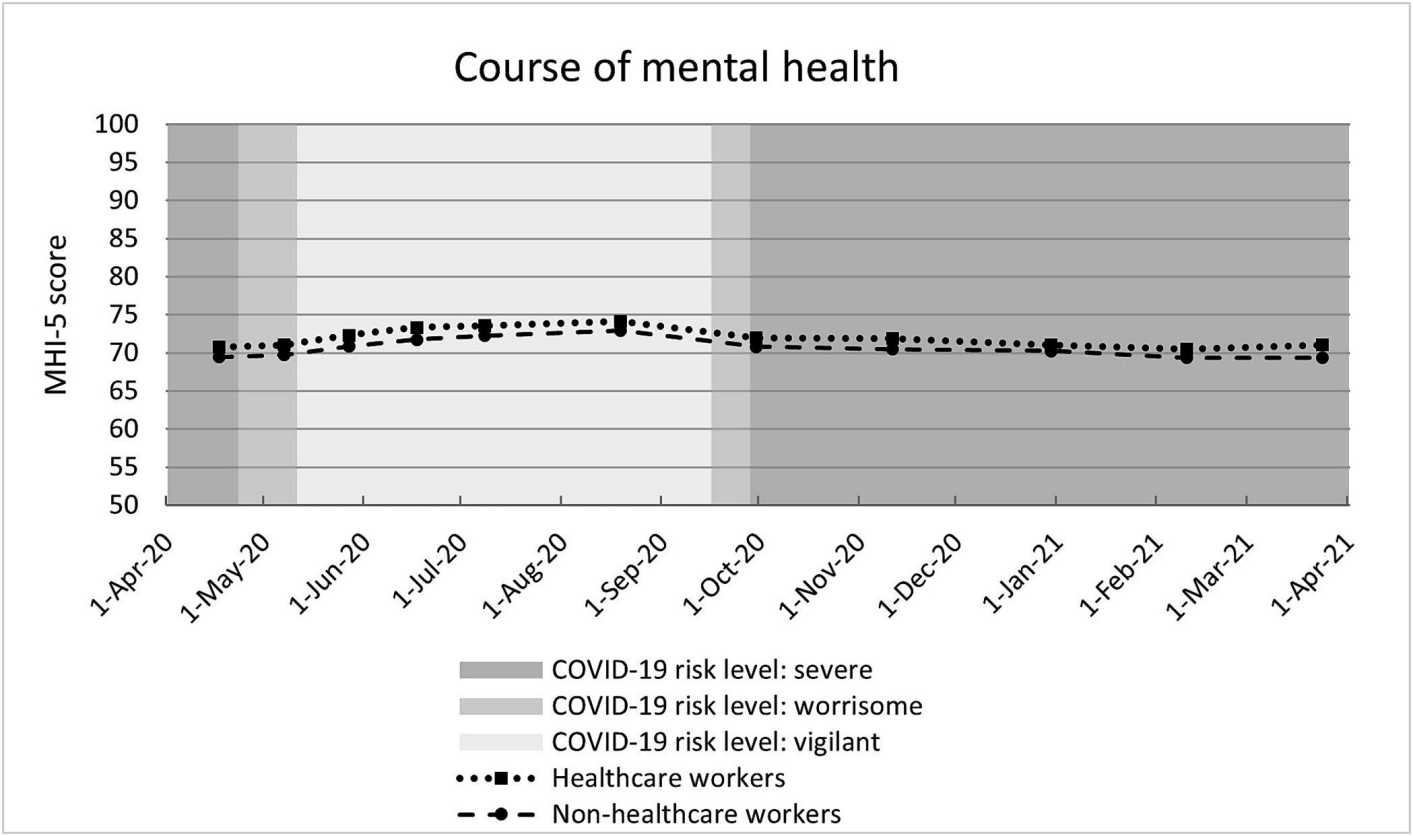
Course of mental health (mean MHI-5 score per round) stratified for healthcare workers and non-healthcare workers. Estimates adjusted for all covariates. NB 1. COVID-19 risk levels: Severe: >100 hospital admissions/day or > 25 ICU admissions/day; Worrisome: 40–100 hospital admissions/day or 10–25 ICU admissions/day; Vigilant: <40 hospital admissions/day and < 10 ICU admissions/day. NB 2. The y-axis ranges from 50 to 100 for visibility purposes (full MHI-5 scale runs from 0 to 100).

The course of the adjusted MHI-5 scores paralleled for both groups, with consistently higher scores among healthcare workers. Mean MHI-5 scores fluctuated throughout the year among both healthcare workers (range 70.4–74.1) and non-healthcare workers (range 69.3–73.0) (Figure 2). Adjusted MHI-5 scores were lower during peak periods of the pandemic, implying poorer mental health at those times.

### Mental health of healthcare workers in different healthcare settings

**Figure 3:**
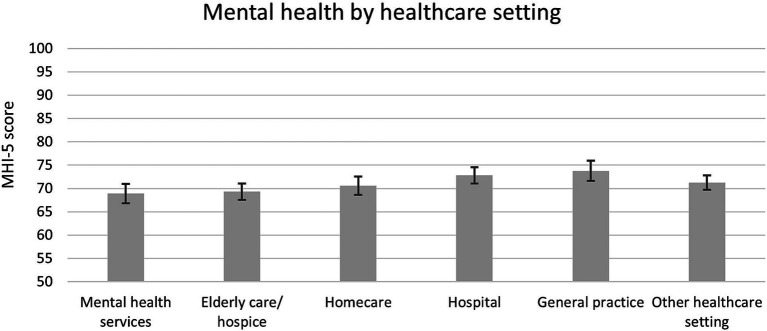
Mental health (mean MHI-5 score with 95%-CI) among healthcare workers during first year of COVID-19 pandemic stratified for healthcare setting. Estimates adjusted for all covariates. NB. The y-axis ranges from 50 to 100 for visibility purposes (full MHI-5 scale runs from 0 to 100).

Healthcare workers who work in mental health services, older adult care or hospice, and homecare were significantly in poorer mental health (adjusted MHI-5 scores 68.9, 69.3, and 70.6, respectively) compared to those working in a hospital and general practice (72.8 and 73.8, respectively), on average during the first year of the pandemic (Figure 3).

## Discussion

During the first year of the COVID-19 pandemic, from April 2020 until March 2021, healthcare workers had a slightly better mental health compared to non-healthcare workers. Mental health fluctuated throughout the year for both groups, with poorer mental health during peak periods of the pandemic, i.e., periods with a higher number of COVID-19 infections and subsequently more restrictive measures to prevent the coronavirus from spreading. Mental health trajectories of healthcare workers and non-healthcare workers had the same course over time and the small difference persisted throughout the year.

The small difference observed (1.29 on a 0–100 scale, 95%-CI = 0.75–1.84) is considered not clinically relevant. Although a relevant difference in MHI-5 scores has not been formally defined, Cohen suggests that a difference in outcome of at least 1/5th (0.2) of the outcome standard deviation implies a small effect size and Norman et al. concludes that a difference in outcome of half of the outcome standard deviation reflects a minimally important difference (27, 28). These cut-off points both exceed our effect size, being 0.08 of the standard deviation in round 11 (SD = 17.2).

It could have been expected that mental health of healthcare workers would have been affected negatively by the pandemic because for this occupational group it is generally not possible to keep the advised distance from patients, which increases infection risk (11). Subsequent health fear, for themselves or their loved ones surrounding them, may lead to excessive stress and mental health problems (12, 13). The results show that healthcare workers did not have poorer mental health compared to non-healthcare workers. These findings are in line with virtually all previous studies comparing healthcare workers with non-healthcare workers, which show that healthcare workers have reported similar or even better mental health outcomes (i.e., stress, anxiety, depression, and PTSD) compared to non-healthcare workers during the first wave of the COVID-19 pandemic (14–17). Our study adds that also general mental health, measured by the Mental Health Inventory-5 (29), did not differ between healthcare workers and non-healthcare workers, and this nondifference persisted throughout the complete first year of the pandemic.

Mental health of healthcare workers fluctuated over time, with poorer mental health during peak levels of the pandemic, which corresponds to findings among Finnish hospital workers (21). In our study the highest score (in August 2020) and lowest score (in February/March 2021) differed 3.7 points among both healthcare workers and non-healthcare workers on the MHI-5 scale. This corresponds to 0.22 of the standard deviation at round 11, which indicates that, compared to a relatively calm period, the peak period of the pandemic had a small negative effect on the mental health of both healthcare workers and non-healthcare workers (>0.20 of SD), although this effect was not clinically meaningful (<0.50 of SD). This is in line with a systematic review, examining the impact of the pandemic by comparing the first months of the pandemic with the pre-pandemic period, which showed that there was an overall increase in mental health symptoms observed in March–April 2020. This review also reveals that mental health of the general population was back at the “normal” level in August 2020 (30). To what extent seasonal influences played a role in the trajectories is not clear, but literature contests a general population shift toward lower mood and more sub-threshold symptoms in spring, autumn or winter (31).

There are several potential explanations for the absence of a relevant difference in mental health during the first year of the COVID-19 pandemic between healthcare workers and non-healthcare workers. A first potential explanation is that mental health of non-healthcare workers has been affected negatively by other aspects of the pandemic than mental health of healthcare workers. One aspect is that a large part of the non-healthcare workers were requested to work from home, which has increased feelings of social isolation (17, 32). A Dutch survey among employees shows that the prevalence of burnout symptoms among home workers increased during peak periods of the pandemic compared to the pre-pandemic period, while the prevalence among location workers remained the same (33). Another aspect that may have affected mental health of non-healthcare workers negatively is the increased job insecurity among employees and self-employed workers (17, 34). Nine percent of employees was afraid to lose their job in the coming 3 months, especially those in the cultural, hospitality and events sectors (35). Over 50% of self-employed workers saw a decrease in the demand for their products or services (36).

Another potential explanation is that, besides the negative mental health effects, there also have been protective factors of the pandemic for healthcare workers. One of these aspects is the finding that healthcare workers feel better informed about the virus and the measures to avoid an infection, and better understand why these measures are needed (14). Also, good psychosocial support, by employers and the community, may have been a protective factor. In some Dutch cities, hospital managers have put together teams of psychologists to support the healthcare workers, and during the first wave of infections, hospital personnel were showered by the community with gifts, flowers and schoolchildren’s drawings (37).

### Strengths and limitations

A strength of this study is that it uses data from a prospective cohort starting from the beginning of the COVID-19 pandemic, which includes a large sample of adults (i.e., 47,254 in Round 11) with representation of all regions of the Netherlands. The longitudinal dynamic design of the survey, with repeated measures of the MHI-5 and high turnover of sent out questionnaires, provides a good indication of how mental health of healthcare workers and non-healthcare workers developed during different stages of the first year of the pandemic.

In the interpretation of the findings, it should also be noted that the CBU cohort is not fully representative: the cohort includes relatively more women, highly educated and people aged 40–60 years compared to the Dutch population. However, for the aim of this study, i.e., examining the association between working in health care and mental health, a representative sample is not required. Among healthcare workers, especially the number of men was low. We considered to exclude male participants from this study, but we checked and determined that gender was not an effect modifier and thus it was methodologically correct to keep male participants in the study sample.

Moreover, from the data it is not clear whether healthcare workers are working directly with COVID-19 patients or not. Multiple studies show that working in the frontline is a risk factor for depression, anxiety, insomnia, distress and trauma-related symptoms (8, 9, 17). It is possible that, due to an immense work-load during the pandemic, the number of frontline workers who found time to participate in the study is relatively low, which could have led to a more positive impression of the mental health of healthcare workers.

Finally, it should be noted that the results only relate to the first year of the pandemic. As the pandemic continued, community support declined while high work demands remained for healthcare workers (38). In later stages of the pandemic, it is possible that another mechanism plays a role, where exhaustion may have an adverse effect on mental health instead of fear of infection. As a result, it is possible that mental health of healthcare workers in later stages of the pandemic deteriorates more strongly compared to the first year.

### Implications policy and research

To further understand what factors played a role in preventing mental health problems among healthcare workers, more research is needed. Regarding hospital workers, it is of interest to examine which factors supported them to maintain good mental health. Considering the poorer mental health among healthcare workers in mental health services, homecare, and older adult care and hospices, it is of interest to examine whether this is related to the pandemic, and if so, what tools (physical/psychological) they lacked and needed during this pandemic to prevent mental health problems. Recent literature shows that during a pandemic, mental health of healthcare workers benefits from informational support, instrumental support, organizational support and emotional and psychosocial support (39). Qualitative research can further identify the needs of healthcare workers within each category and in each workplace setting. These insights are useful to respond to in order to maintain good mental health among healthcare workers during another pandemic.

## Conclusion

During the first year of the COVID-19 pandemic, there was no relevant difference in mental health between healthcare workers and non-healthcare workers in the Netherlands. During peak periods of the pandemic, mental health of both healthcare workers and non-healthcare workers was poorer. To be better prepared for another pandemic, future research should reveal which factors hindered and which factors supported healthcare workers to maintain good mental health.

## Data availability statement

The datasets presented in this article are not readily available because the AVG (‘Algemene Verordening Gegevensbescherming’) data cannot be shared publicly, unless aggregated. For academic collaborations and publishing in scientific journals, a Behavioral Science Consortium (‘Be-Prepared’) has been initiated with researchers working at universities as well as the RIVM. Requests to access the datasets should be directed to coronagedragsunit@rivm.nl.

## Ethics statement

Ethical approval was not required for the studies involving humans because the CBU cohort study does not meet the requirement as laid down in the Law for Research Involving Human Subjects (WMO) and was therefore exempted by the Centre for Clinical Expertise at RIVM from formal ethical review (Study number G&M-561). The studies were conducted in accordance with the local legislation and institutional requirements. The participants provided their written informed consent to participate in this study.

## Author contributions

MN, SO, KP, BL, and CB contributed to the study conception and design. MB and FK coordinated the data collection. MN performed the analysis and wrote the first draft of the manuscript. All authors commented on previous versions of the manuscript, read, and approved the final manuscript.
