# Supplementary material for: Mental health of healthcare workers during the first year of the COVID-19 pandemic in the Netherlands: a longitudinal study
Source: Front Public Health. 2023 Nov 23;11:1224112. doi: 10.3389/fpubh.2023.1224112 (PMC10701889; doi:10.3389/fpubh.2023.1224112)
Supplement: Supplementary file 1 [file Data_Sheet_1.docx]

Appendix A

Construction of covariates

Age was categorized in ten-year categories, except for the youngest age category (18-29 years old). Highest completed educational level was originally measured in eight categories, which was subsequently categorized into low, middle and high educational level, in line with the classification of Statistics Netherlands [1]. Having a (chronic) health condition was measured with the item: ‘Do you have one or more of the following conditions: chronic respiratory or lung problems, chronic heart disease, diabetes mellitus, severe kidney disease leading to dialysis or kidney transplantation, an HIV infection, severe liver disease, obesity (BMI>40) or lower resistance to infection due to various reasons?’. Response options were yes or no. Past suspected/confirmed COVID-19 infection was categorized into yes and no. Suspected infection was also included, as this situation was particularly common in the early stages of the pandemic when testing facilities were generally not available. Household composition was measured as living alone, living with partner, living with children ≤12 years, living with children >12 years and living with others. Respondents who were living with both children aged ≤12 years and >12 years were categorized as living with children ≤12 years. Experienced quality of social contacts was measured with the item: ‘Looking back at the past 7 days, to what extent do you disagree or agree with the following statement: All in all, I experience the quality of my social contacts to be good.’ Response categories ‘strongly disagree’ and ‘disagree’ were categorized into ‘not good’, ‘neutral’ into ‘neutral’ and ‘agree’ and ‘completely agree’ into ‘good’.

Reference

1. Statistics Netherlands. *Education level*. 2018; Available from: <https://www.cbs.nl/en-gb/news/2018/20/well-being-not-distributed-equally/education-level>.
